# Epidemiology and Management Trends of Patients With Sepsis and Septic Shock in the Intensive Care Unit: A Prospective Trial in the Caribbean

**DOI:** 10.7759/cureus.10980

**Published:** 2020-10-16

**Authors:** Keevan Singh, Seetharaman Hariharan, Dale Ventour, Deryk R Chen, Lorna G Merritt-Charles, Mark Sookwah, Daynish Maharaj, Sasha Sankar-Maharaj

**Affiliations:** 1 Anaesthesia and Intensive Care, University of the West Indies, St Augustine, TTO; 2 Anaesthesia and Intensive Care, San Fernando General Hospital, San Fernando, TTO; 3 Anaesthesia and Intensive Care, Eric Williams Medical Sciences Complex, Mt. Hope, TTO; 4 Anaesthesia and Intensive Care, Port of Spain General Hospital, Port of Spain, TTO; 5 Anaesthesia and Intensive Care, Sangre Grande Hospital, Sangre Grande, TTO

**Keywords:** sepsis, septic shock, icu, sofa, ards, aki, outcomes, mortality

## Abstract

Objectives

To investigate the epidemiology, management, and predictors of mortality in severe sepsis and septic shock in the intensive care units (ICUs) of Trinidad, Trinidad & Tobago.

Methods

A prospective observational study in four ICUs over a one-year period (August 2017-August 2018) was conducted. Physiologic variables, treatment data, and outcomes were collected on admission to ICU and daily until 28 days. The 28-day mortality and ICU mortality were recorded. Subgroup analysis was performed based on survival, and predictors of mortality were determined through logistic regression.

Results

Outcome data were available for 163 patients. The 28-day mortality rates for sepsis and septic shock were 42% and 47%, respectively. ICU mortality rate for sepsis was 34%. The most common suspected source of infection was pneumonia (33%). Acute kidney injury (AKI) was common and present in 71% of patients, with renal replacement therapy only being used in 30% of cases. Mechanical ventilation was required in 84% of cases. Moderate-to-severe acute respiratory distress syndrome (ARDS) (OR: 4; 95% CI: 1.9-8.8; p < 0.001) and the development of AKI (all stages) (OR: 10; 95% CI: 3.9-30.2; p < 0.001) were found to be predictive of mortality. Incidence of mechanical ventilation, moderate-to-severe ARDS, stage 3 AKI, septic shock, and failure to achieve a mean arterial pressure of > 60 mmHg within the first 24 hours of admission were higher in patients who did not survive (p < 0.05).

Conclusions

Sepsis and septic shock are associated with a high 28-day mortality. Organ dysfunction with renal and pulmonary involvement was an important factor in predicting a higher mortality.

## Introduction

Over the last decade, there have been several innovations in the diagnosis and management of sepsis and septic shock [1,2]. Current definitions focus on the detection of organ dysfunction instead of inflammatory parameters, with sepsis now defined as a dysregulated host response rather than a purely inflammatory condition [2]. However, despite these advances, there remains a significant global burden of disease, with 49 million cases of sepsis and 11 million deaths being reported worldwide in 2017 [3].

Stevenson et al. using control arm data from randomized clinical trials showed a mortality rate of up to 30% for severe sepsis [4]. In developing and low-resource countries, which may not have been represented by trial data, the mortality rate for sepsis is even higher, with an almost 50% mortality rate being reported for severe sepsis and septic shock in developing and resource-poor countries [5-8].

The epidemiology of sepsis has been well studied in developed countries, with most guidelines being developed based on these populations [8]. Sepsis in low-resource settings has its own unique epidemiological and therapeutic challenges, hence the need for a more global focus in sepsis [9]. As sepsis remains a significant global disease, data from developing and low-resource countries can add global insight into the study of this disease process.

Investigators from many resource-limited countries have published data regarding the epidemiology and outcomes of sepsis in their regions [3-5]. In a single emergency department cohort in Jamaica, where 117 patients with severe sepsis and septic shock were studied, a mortality rate of 25% was observed [10]. To date, there has been no consistent published data on sepsis and sepsis outcomes in a Caribbean intensive care unit (ICU) setting.

Trinidad & Tobago is a twin-island republic lying close to the South America mainland with a population of approximately 1.5 million and a diverse ethnic population. Although ranked as a high-income country according to the World Bank, there are currently only two adult ICU beds per 100,000 persons locally. In developing countries, such as ours, prioritized healthcare spending can present problems for critically ill patients. Globally, many patients with sepsis and septic shock require ICU level care. Hence, the burden of sepsis on countries with low ICU bed numbers is significant. This can thus pose significant public health concerns to healthcare resource-poor countries.

The aim of our study is to investigate the 28-day mortality rate of severe sepsis and septic shock in Trinidadian ICUs. The severity scores, location of the septic source, treatment trends, and factors associated with mortality will also be described in our population.

## Materials and methods

This study was conducted as a prospective observational trial in the four local ICUs, San Fernando General Hospital (SFGH), Eric Williams Medical Sciences Complex (EWMSC), Port of Spain General Hospital (POSGH), and Sangre Grande Hospital (SGH). The trial was run over a one-year period from August 2017 to August 2018.

Approvals were obtained from the Campus Research Ethics Committee, The University of the West Indies, St Augustine, and the Ethics Committees of each of the four participating hospitals. The respective ethics committees, due to the observational nature of the study, waived requirements for written informed consent. Data collected were limited to only that which formed part of the routine clinical management of intensive care patients.

Patients

All patients older than 18 years admitted to the ICU and fitting the criteria for sepsis and septic shock during the study period were eligible for inclusion.

Exclusion criteria included patients who presented with a primary diagnosis of hemodynamic instability secondary to ongoing bleeding, burns, acute coronary syndrome, acute major cerebrovascular event, acute pulmonary edema, seizures, drug overdose, and major cardiac arrhythmias.

Definitions

Sepsis was defined according to the Third International Consensus Definition for Sepsis and Septic Shock (Sepsis-3): suspected or confirmed infection and a change in Sequential Organ Failure Assessment (SOFA) score ≥ 2 [2]. Patients who were not known to have any organ dysfunction were listed as having a baseline SOFA of 0. Septic shock was diagnosed in patients with sepsis and persistent hypotension requiring vasopressors to maintain a MAP of ≥65 mmHg, despite adequate fluid resuscitation and evidence of tissue hypoperfusion. As lactate was not readily available, base excess ≤ 4mmol/L was used as evidence of tissue hypoperfusion when diagnosing septic shock [2,11,12].

Acute respiratory distress syndrome (ARDS) was diagnosed according to Berlin criteria, and KDIGO (Kidney Disease Improving Global Outcomes) criterion was used for diagnosis and staging of acute kidney injury (AKI) [13,14].

Data collection

All ICU admissions were screened by team members at the various hospitals for possible inclusion. Data were obtained from the patients’ notes using a manual data collection instrument. This instrument was frequently inspected by the individual clinical leads to ensure the accuracy and completeness of data entry. Data collected included demographics, suspected source of sepsis, monitoring used, management trends, and outcome data that included length of stay in ICU, days on mechanical ventilation, and survival. Clinical data were collected for calculating maximum SOFA and Acute Physiology and Chronic Health Evaluation II (APACHE II) scores in the first 24 hours of admission to the ICU. Mean arterial pressure (MAP) was recorded hourly for the first 24 hours and used to determine the mean MAP over the first 24 hours. Patients were also followed daily during their ICU stay for the development of ARDS and AKI. Mortality was recorded in ICU as well as at 28 days after ICU admission.

Analysis

Data analysis was performed using Statistical Package for Social Sciences (SPSS) Version 20 (IBM Corp., Armonk, NY, USA). Normally distributed data were presented as mean and standard deviations (SD), data which were not normally distributed were presented as median and interquartile ranges (IQRs), and categorical data were presented as proportions. Continuous normally distributed data were analyzed using Student’s t-test and non-normally distributed data were analyzed using the Mann-Whitney U test. Categorical data were analyzed using the chi-square or Fisher exact test, as appropriate.

Multivariate logistic regression was used to determine the factors associated with 28-day and ICU survival. Predictors were chosen based on their reported predictive ability, use in other predictive models, current clinical usage, and perceived importance. The predictor and outcome variables were analyzed with a stepwise approach using a backward elimination method based on the likelihood ratio (backward LR). Mortality predictors used in the regression analysis included APACHE II and SOFA scores, age, MAP < 60 mmHg within the first 24 hours, base excess, presence of septic shock, days on mechanical ventilation, and development of moderate-to-severe ARDS or AKI during their ICU course. A p-value of <0.05 was considered statistically significant.

## Results

On admission to ICU, 183 patients were suspected of having severe sepsis. Out of these, 163 persons met the inclusion criteria and were included in the study. Outcome data were available for all 163 persons, and they were included in the analysis.

Demographics

The overall demographics are presented in Table 1.

**Table 1:** Demographic factors of study population *p-Values were derived using chi-square analysis, Fisher exact test, and Mann-Whitney test as appropriate. IQR, interquartile range

Demographics	All patients (n= 163)	Survivors (n = 95)	Non-survivors (n = 68)	p-Values*
Male, n (%)	82 (50)	37 (47)	45 (54)	0.43
Female, n (%)	81 (50)	50 (53)	31 (38)	0.43
Age, median (IQR)	52 (39-65)	50 (39-61)	54 (40-71)	0.07
Ethnicity, n (%)				
Afro-Trinidadian	43 (26.4)	24 (25)	19 (28)	0.72
Indo-Trinidadian	108 (66.3)	66 (70)	42 (62)	0.32
Mixed/other	12 (7.4)	5 (5)	7 (10)	0.24
Hospital, n (%)				
Hospital 1	101 (62)	41 (60)	60 (63)	0.745
Hospital 2	26 (26)	17 (17.9)	9 (13.2)	0.52
Hospital 3	20 (20)	9 (9.5)	11 (16.2)	0.23
Hospital 4	16 (9.8)	9 (9.5)	7 (10.3)	0.86

Gender was evenly split, with males and females consisting of 50% of the population. Persons of East Indian descent (Indo-Trinidadian) comprised the largest proportion of patients (108 [66%]) followed by those of African descent (Afro-Trinidadian) (43 [26%]). The median age of the population was 52 years (range: 39-65 years). The majority of cases came from one hospital, which accounted for 101 (62%) cases.

Characteristics

The descriptive clinical variables are depicted in Table 2.

**Table 2:** Patient characteristics and sepsis-related organ involvement ARDS, acute respiratory distress syndrome; APACHE II, Acute Physiology and Chronic Health Evaluation II; AKI, acute kidney injury; AKIN, Acute Kidney Injury Network; CNS, central nervous system; ICU, intensive care unit; IQR, interquartile range; MAP, mean arterial pressure, RRT, renal replacement therapy; SOFA, Sequential Organ Failure Assessment

Characteristics median (IQR)	All patients (n = 163)	Survivors (n = 95)	Non-survivors (n = 68)	p-Values
SOFA	8 (6-10)	8 (5-10)	9 (7-1)	0.03
APACHE II	17 (13-22)	15 (12-20)	20 (16-27)	<0.001
Mechanically ventilated (%)	137 (84)	73 (77)	64 (94)	<0.001
Base excess	-9 (-12 to -5)	-8 (-11 to -5)	-11 (-13 to -6)	<0.001
AKI (any stage), n (%)	115 (71)	53 (56)	62 (91)	<0.001
AKIN stage 1	7 (4.3)	3 (3)	4 (6)	0.45
AKIN stage 2	31 (19)	15 (15.8)	16 (24)	0.23
AKIN stage 3	77 (47)	35 (37)	42 (62)	<0.001
RRT (%)	48 (30)	23 (48)	25 (52)	0.08
Moderate-to-severe ARDS (%)	74 (45)	32 (43)	42 (57)	<0.001
Septic shock (%)	119 (73)	63 (66)	56 (82)	0.03
MAP < 60 mmHg (%)	12 (7.4)	2 (2)	10 (15)	0.00
Days on ventilator	4 (1-8)	4 (1-7)	5 (2-11)	0.02
ICU days	6 (3-11)	6 (4-10)	6 (3-13)	0.96
Suspected source (%)				
Lungs	54 (33)	34 (36)	20 (29)	0.40
Urological	23 (14)	19 (20)	4 (6)	0.01
Abdomen	27 (17)	10 (11)	17 (25)	0.02
Pancreas	10 (6)	6 (6)	4 (6)	1.00
CNS	6 (4)	2 (2)	4 (6)	0.23
Skin/soft tissue	9 (5)	5 (5)	4 (6)	1.00
Surgical site	5 (3)	1 (1)	4 (6)	0.16
Other/unknown	29 (18)	18 (19)	11 (16)	0.68

Median (IQR) admission SOFA and APACHE II scores were 8 (6-10) and 17 (13-22), respectively. The majority of patients, 119 (73%), had septic shock. A total of 115 (71%) patients developed AKI, of which 77 (43%) developed stage 3 AKI. Renal replacement therapy (RRT) was only used in 48 (30%) patients. Moderate-to-severe ARDS was present in 74 (45%) patients, and the respiratory tract (lungs) was found to be the most commonly affected source of infection (54 [33%]). Mechanical ventilation was required in 137 (84%) patients. 

No specific source could be found in 18% of patients. An abdominal and urological source was found in 17% and 14% of patients, respectively.

Management parameters

The management variables are shown in Table 3.

**Table 3:** Management parameters of patient population CVP, central venous pressure; MAP, mean arterial pressure

Management parameter, n (%)	N = 163
Arterial line	160 (98)
CVP line	147 (90)
Blood culture within 24 hours	139 (85)
Average MAP > 60 mmHg within 24 hours	152 (93)
Positive culture obtained	21 (13)
Vasopressor choice	
None	41 (25)
Noradrenaline	69 (42)
Dopamine	3 (2)
Noradrenaline and adrenaline	33 (20)
Noradrenaline and dobutamine	3 (2)
Other	14 (9)
Antibiotic choice	
Meropenem/imipenem	41 (25)
Piperacillin-tazobactam	54 (33)
Third-generation cephalosporin (ceftriaxone, cefuroxime, cefotaxime)	35 (22)
Levofloxacin	7 (4)
Other (monotherapy)	7 (4)
Combination therapy	19 (12)

Most patients were managed with arterial (98%) and central lines (90%). A mean MAP of > 60 mmHg in the first 24 hours of admission was achieved in 152 (93%) patients. Noradrenaline was the most common vasopressor used in 69 (42%) patients, and this was followed by the noradrenaline-adrenaline combination in 33 (20%) patients (Table 3). Positive cultures were only obtained in 21 (13%) patients. *Escherichia coli *was the most common organism cultured (19%) followed by *Acinetobacter *(14%) and *Klebsiella* (14%). Piperacillin-tazobactam was used in 33% of patients followed by carbapenems (meropenem/imipenem) in 25%.

Outcomes

The ICU mortality rate was 34%, whereas the 28-day mortality rate for sepsis was 42%. Patients with septic shock had a 28-day mortality rate of 47%. Patients spent a median of 6 days (range: 3-11 days) in the ICU with 4 days (range: 1-8 days) on a mechanical ventilator. There was no statistical difference in outcomes based on admitting hospital.

Comparison based on 28-day survival

The variables were compared between survivors and non-survivors at 28 days, which is shown in Table 2. Non-survivors had significantly higher SOFA (p=0.03) and APACHE II (p < 0.001) scores on admission than survivors. Base excess was significantly lower in non-survivors (p < 0.001). The mortality was also higher in patients requiring mechanical ventilation compared to those who did not require this intervention (94% vs 77%, p < 0.001). Incidence of moderate-to-severe ARDS, stage 3 AKI, septic shock, and a failure to achieve a mean MAP of >60 mmHg was significantly higher in patients who did not survive (p < 0.05). Urological sepsis was more common in survivors (p = 0.01), whereas abdominal sepsis was more common in patients who died (p = 0.02).

Comparison based on ICU survival

Significant differences between groups were similar to trends seen in 28-day survival (Table 2). APACHE II score, SOFA score, base excess, stage 3 AKI, moderate-to-severe ARDS, septic shock, and urological and abdominal sepsis were all significantly different between ICU survivors and non-survivors. The incidence of RRT was higher in patients who died in ICU (41% vs 23%; p = 0.02).

Predictors of 28-day mortality

In the logistic regression analysis (Table 4), out of the initial predictors and using the backward LR approach, three variables (any stage of AKI, moderate-to-severe ARDS, and low MAP) were included as the final predictors for 28-day mortality and four variables for ICU mortality (any stage AKI, moderate-to-severe ARDS, low MAP, base excess).

**Table 4:** Predictor variables and its effect on 28-day outcome in the logistic regression analysis *Represents the variables that were included in the final analysis using the backward LR method. ARDS, acute respiratory distress syndrome; AKI, acute kidney injury; LR, likelihood ratio; MAP, mean arterial pressure; SOFA, Sequential Organ Failure Assessment

Predictor variable for 28-day outcome	OR (95% CI)	p-Value
Apache II	1.1 (0.98-1.10)	0.16
SOFA score	0.9 (0.70-1.06)	0.22
Age	1.0 (0.97-1.00)	0.96
Ventilator days	1.0 (0.97-1.10)	0.30
Septic shock	1.0 (0.30-2.95)	0.92
Base excess	1.0 (0.88-1.05)	0.41
Moderate-to-severe ARDS^*^	4.0 (1.89-8.83)	<0.001
Low MAP^*^	6.1 (0.94-40.33)	0.06
Any AKI^*^	10.9 (3.93-30.17)	<0.001

In the logistic regression analysis, the only factors that were significantly associated with 28-day mortality were the presence of moderate-to-severe ARDS (OR: 4; 95% CI: 1.9-8.8; p < 0.001) and the development of AKI (any stage, OR: 10; 95% CI: 3.9-30; p < 0.001). AKI and moderate-to-severe ARDS were also associated with ICU mortality (p < 0.01).

## Discussion

Sepsis has received considerable attention over the last decade with an attendant increase in awareness of its presentation and need for emergent management. Global initiatives such as the Surviving Sepsis Campaign (SSC) have sought to improve the quality of care for these patients by advocating protocolized care and implementing evidence and best practice-based recommendations to guide clinicians in the early diagnosis, monitoring, and management of the disease [1]. Adherence to these recommendations and performance bundles, even in low-resource and middle-income countries, has been shown to reduce the odds of hospital mortality [15].

When the management parameters in the patients of the present study are considered (Table 3), it can be noted that the majority of patients had arterial and central venous pressure monitoring and more than 90% were able to obtain a MAP > 60 mmHg within the first 24 hours. Empiric broad-spectrum antibiotics were also frequently used, and a blood culture was performed within 24 hours in 85% of patients. Noradrenaline was also the most commonly used vasopressor (42%). These findings illustrate that clinicians are aware of the standard management guidelines for sepsis. However, the challenge exists in translating this knowledge to consistent practice to improve outcomes, which from time to time may be significantly limited in a resource-constrained healthcare system. We also note that only 13% of patients had positive cultures, and this may have been due to the fact that most cultures were taken late and after antibiotics were started.

After the original paradigm shift from the initial SSC, subsequent randomized controlled trials in the United States, United Kingdom, and Australia have shown no difference between this protocolized care and routine care groups [16-18]. However, all the standard care groups received a high level of care including prompt resuscitation similar to that advocated by SSC. Despite this foundation for clinical management of sepsis, the challenge still exists in knowing which practices are feasible and cost-efficient to adopt in low-resource countries [9].

The median age of the patient cohort in the present study was 52 years, which represented a relatively younger cohort of affected patients compared to those of more developed countries, where patients generally tend to have been a decade older [16,18]. Healthcare initiatives in this patient group can have a meaningful impact on society and may justify an increased critical care spending to improve outcomes. Loss of these relatively younger patients from sepsis in low-resource settings can have a far-reaching economic impact on the affected families [9].

Shortages of ICU beds are a significant problem in sepsis management. This arises due to the intensity of management advocated in guidelines and the need for organ support, which can only be found in ICUs. Sufficient ICU beds in many low-resource countries pose a dilemma especially when there may be other pressing healthcare needs [9]. The majority (84%) of the patients in this study needed mechanical ventilation and 73% required vasopressor infusions, making ICU admission and critical care management mandatory. In comparison, a study published from Jamaica, another Caribbean country, showed that although 113 patients were diagnosed to have severe sepsis in the emergency department, only one patient could be admitted to the ICU [10]. This highlights the ICU-bed deficiencies plaguing low-resource settings.

The median admission SOFA and APACHE II scores were 8 (range: 6-10) and 17 (range: 13-22), and the overall predicted mortality was 33% and 25%, respectively. This may indicate that the intensity of the illness was severe in this cohort of the patients on presentation to the ICU. This was comparable to the severity of illness scores reported in many recently reported sepsis trials where admission SOFA scores ranged from 2 (United Kingdom) to 8 (Japan, India) and APACHE II scores ranged from 15 (Australia) to 21 (United States) [7,16-19].

The 28-day mortality rate for sepsis and septic shock was 42% and 47%, respectively, and both these are comparatively higher than those reported in recent trials. However, many of these reports were from high-income developed countries, and the mortality (60 and 90 days) ranged from 18-30% [16-18]. When considering global data, the in-hospital mortality rate due to sepsis ranges from 40 to 60% [8]. In the present study, there was also a notable difference between ICU mortality (34%) and 28-day mortality. It is likely that after discharge from ICU to the general wards, the level of care provided to such patients was not commensurate with the unique challenges faced by such patients. This could have led to an increase in mortality as seen. Low ICU bed numbers would also increase the pressure on clinicians for early discharge in a patient who may still require a higher level of care than could be provided in the general wards. Quality improvement measures in sepsis for low-resource countries should aim to provide a consistent level of care for these patients, not only in the emergency departments and ICUs but also in general medical and surgical wards. Failure to do this can lead to wasted resources spent in the ICU, with no overall beneficial impact of ICU care on patient mortality.

The presence of moderate-to-severe ARDS (OR = 4) and the development of any stage AKI (OR = 10) were significantly associated with 28-day mortality in the present study. Both these conditions require significant critical care expertise, and in the case of AKI, additionally, significant resources are needed. This can be inferred from the fact that AKI was diagnosed in 71% of patients, whereas only 30% received RRT. The sole modality used for RRT was hemofiltration, which was only performed in one ICU of all the four ICUs studied. The high cost of replacement fluids is an important consideration in this regard. Poor access to RRT in patients with AKI is a recognized problem and has been reported from low- and middle-income countries [20].

Pneumonia was the most commonly identified source of sepsis (33%), with gram-negative organisms being the most commonly cultured. Almost half of all the study patients developed moderate-to-severe ARDS (45%). ARDS was a 28-day mortality predictor in the present study, which follows the trend of high global ICU mortality rate associated with ARDS. In the LUNG-SAFE (Large observational study to UNderstand the Global impact of Severe Acute respiratory FailurE) study using an international cohort, moderate and severe ARDS was associated with a mortality rate of 40% and 46% respectively, similar to that of the present study [21]. The authors noted that ARDS was largely unrecognized and identified several shortcomings in clinical management, which could have been true in our setting also.

There were several limitations to our study. The total number of patients admitted to the ICUs during the period was not recorded, and hence the overall incidence of sepsis could not be determined. One ICU represented 63% of cases; hence, there is a possibility that the incidence of sepsis might have been under-reported in the other ICUs. No data were available on patients’ conditions before ICU admission and the lead-time from the identification of sepsis to ICU admission, which may have further affected the outcomes.

Although lactate is generally regarded as the gold standard in diagnosing hypoperfusion, due to its limited local availability, base excess was used as a surrogate for lactate when septic shock was diagnosed [11]. This may have led to some patients' misclassification as septic shock due to the imperfect relation between lactate and base excess. However, the majority of patients classified as septic shock (97%) were on moderately high doses of vasopressors, making the diagnosis likely.

## Conclusions

In conclusion, this study was able to describe the epidemiology and management trends for sepsis in four ICUs in Trinidad. Sepsis and septic shock are associated with high mortality rates, and mortality is also higher after discharge from ICU. This study will provide basal data from a Caribbean country regarding sepsis and its management, which will be useful for quality improvement measures including resource allocation.
